# Victimisation in the life of persons with severe mental illness in Uganda: a pluralistic qualitative study

**DOI:** 10.1186/s12888-024-05720-4

**Published:** 2024-04-30

**Authors:** Rwamahe Rutakumwa, Birthe Loa Knizek, Christine Tusiime, Richard Stephen Mpango, Carol Birungi, Eugene Kinyanda

**Affiliations:** 1 Medical Research Council, Uganda Virus Research Institute & London School of Hygiene and Tropical Medicine Uganda Research Unit, Plot 50-59 Nakiwogo Road, P. O. Box 49, Entebbe, Uganda; 2 Department of Mental Health, Norwegian University of Science and Technology, Trondheim, Trøndelag Norway; 3 Butabika National Referral Mental Hospital, Old Port Bell Road, P. O. Box 7017, Kampala, Uganda; 4 Department of Psychiatry, College of Health Sciences, Makerere University, Kampala, Uganda; 5 Department of Psychiatry, Makerere University, P. O. Box 7072, Kampala, Uganda

**Keywords:** Victimisation, Abuse, Severe mental illness, Uganda, Sub-saharan Africa

## Abstract

**Introduction:**

Victimisation of persons with severe mental illness is recognised as an urgent global concern, with literature pointing to higher rates of violent victimisation of persons with severe mental illness than those of the general population. Yet, for low income countries, there is a huge gap in the literature on the risk, character and victims’ in-depth experiences of victimisation of persons with severe mental illness. We explore the lived experiences and meanings of victimisation of persons with severe mental illness in Uganda, and discuss their implications for care of the mentally ill.

**Methods:**

A pluralistic qualitative study was undertaken to explore victimisation among patients with severe mental illness. Patients who had suffered victimisation were purposively sampled from Butabika National Referral Mental Clinic and Masaka Regional Referral Hospital, following confirmation of symptom remission. In-depth interviews were held with 18 participants, comprising 13 females and 5 males from low to moderate socioeconomic status. Interpretative phenomenological analysis and thematic content analysis were conducted.

**Results:**

Victimisation was exhibited in three main forms: (a) psychological, expressed in attitudes towards mentally ill family members as valueless and dispensable, and stigmatisation, (b) physical, as manifested in beatings, indoor confinement and tethering mostly by family members and (c) sexual victimisation, particularly rape. Also observed were victim’s various responses that pointed to the negative impact of victimisation, including a heightened risk of suicide, social withdrawal, a sense of hatefulness and a predisposition to more victimisation.

**Conclusion:**

The family environment plays a predominant role in perpetrating victimisation of the mentally ill in some sub-Saharan African contexts such as Uganda. We propose a holistic framework for mental health interventions, incorporating biomedical but notably also social determinants of mental health, and targeted at improving familial relationships, social support and a sense of belongingness both within the family and the broader community.

## Introduction

Victimisation of persons with severe mental illness (including bipolar disorder, schizophrenia and other psychotic disorders) has been recognised as an urgent global concern [1], with a review of literature unequivocally pointing to higher rates of violent victimisation of persons with severe mental illness than those of the general population [2–9]. In a Dutch study, for example [4] the annual rate of prevalence of victimisation of outpatients with severe mental illness was 47% as compared to 32% of the general population. In a Greek study [10], 59% of persons living with severe mental illness reported having been victimised as compared to 46% of healthy controls. In the context of low and middle income countries (LMICs), there is a huge gap in the literature about the risk of violent victimisation of persons with severe mental illness [11]. Even so, findings from the few studies conducted in this region have been consistent with those from high income countries. For example, in an Ethiopian study, the prevalence rate of violent victimisation among persons with severe mental illness was 61% against 42% for those without the illness [11].

Certain risk factors have been identified in explaining predisposition to victimisation of persons with severe mental illness, including substance abuse, symptom severity, young age, unemployment, criminal history and homelessness [12, 13]. Also well documented are the effects of victimisation, with the vice having been found to adversely impact the course of mental illness in the long term, further diminishing the quality of life of persons with severe mental illness and their families [2, 14, 15]. Relatedly, victimisation has been cited in exacerbating distress among the mentally ill [10, 12] thereby aggravating the patients’ mental illness [12, 16]. Furthermore, abuse of the mentally ill has been known to escalate the cost of healthcare and demand for services, putting a strain on the healthcare system [17], a phenomenon impacting sub-Saharan Africa even more given that mental health services in the region are still underdeveloped [18–20].

In spite of the apparently large volume of literature on victimisation of persons with severe mental illness, gaps in research on the subject still exist. First, much of the work on this subject has been conducted in high income countries, hence little is known about the situation in LMICs, where the sociocultural, structural and legal conditions impacting the risk of violence differ markedly from those in high income countries [11]. Indeed, this dearth of literature on LMICs was underscored by Tsigebrhan and colleagues [11] who as of 2014 were able to identify only one study in sub-Saharan Africa that focused on violent victimization of the mentally ill. Our current review of literature suggests very little change since then. Second, a lot more attention in these studies has been on persons with severe mental illness as perpetrators than as victims of the abuse [10, 21, 22]. Third, our review of literature suggests that majority of the publications are from studies using quantitative methods, implying that victims’ deeper experiences and interpretations of abuse, especially in the sub-Saharan African context, remain largely unexplored. Yet, this knowledge of the victims’ “life-world” [23] – that is, the way the phenomenon of victimisation appears to those experiencing it – is critical for designing fully informed, contextually appropriate interventions for addressing this important social problem. Fourth, literature has been scanty on the profile of abusers and how they relate to the victim [13].

Our purpose in this paper was to explore the lived experiences and meanings of victimisation of persons with severe mental illness in Uganda, including the history and the typical abuser, and to discuss their implications for care of the mentally ill.

## Methods

### Design

This was a pluralistic qualitative sub-study to explore victimisation among patients with severe mental illness. The sub-study was an offshoot of a bigger project comprising two studies: (i) the Main Study which investigated the epidemiology of HIV infection and risky sexual behaviour among patients with severe mental illness (SMI) and (ii) the Clinical Trials Preparedness study which examined the feasibility and acceptability of undertaking clinical trials among patients with comorbid SMI/HIV [24, 25].

### Study setting

The study was conducted at Butabika National Psychiatric Referral Hospital and the Mental Health section of Masaka Regional Referral Hospital. Butabika Hospital is the only tertiary referral facility for psychiatric care in Uganda and handles patients from all over the country. In addition to the general psychiatric service, the hospital has specialised psychiatric units such as the forensic unit, alcohol and substance abuse unit, the child and adolescent unit and an infectious disease clinic (IDC clinic) that manages patients with comorbid SMI/HIV. Butabika Hospital also provides general outpatient services to the community through a daily out-patient clinic. Masaka Regional Referral Hospital, on the other hand, offers all services expected of a regional referral health facility, including psychiatric services for both adults and children. Psychiatric services at the Mental Health section of the hospital are provided by a team of psychiatric clinical officers and psychiatric nurses who receive quarterly support supervision from Butabika tertiary referral hospital.

### Sampling

The sample for this sub-study was drawn from a large pool of 1,201 participants who had been recruited for the Main Study and were attending mental health clinics at Butabika National Referral Mental Clinic and Masaka Regional Referral Hospital. The initial sample for the qualitative sub-study was purposive, comprising 409 (of the 1,201) participants who had met criteria for ‘ever suffered adulthood physical abuse’ and 263 participants from the same large sample who had ‘ever suffered adulthood sexual abuse’. In both cases, the participants had suffered the abuse before they acquired a mental illness or while they suffered a mental illness or both. From the purposive sample totalling 672 participants above, 18 participants were selected for the qualitative interviews on the day of their visitation at the mental health clinic following confirmation of symptom remission by an attending psychiatrist or psychiatric clinical officer. Selection of the participants was done in such a way as to ensure that the sample was fairly balanced in featuring both those with a history of physical and of sexual abuse. The total number of interviewees was determined when it became apparent that additional interviews were not yielding new information. No attempt was made to interview the primary caregiver, as this was not provided for in the study design.

### Data collection procedures

In-depth interviews were conducted with the 18 participants reporting victimisation, which had been categorised as either physical or sexual. An interview guide was used to collect data. The guide was developed by the researcher who brainstormed and listed questions and topics based primarily on the study’s research questions, but also on scholarly literature on related previous work. Informed consent was obtained at least one week after individuals received the study information sheet in order to allow them sufficient time to read and internalise the content. Participants were interviewed at the health facility where they received care or any other venue of their choice by a trained research assistant who was either a psychiatric nurse or a clinical psychologist. The interview explored events leading to victimisation, how it occurred, the abuser, and the impact of such victimisation on the mental health of the participant. All the interviews were voice-recorded, upon obtaining consent from the respective participants.

### Data management and analysis

Data were transcribed verbatim by the research assistant who conducted the interview and stored the data according to the MRC guidelines. We adopted a pluralistic analytical approach that combined interpretative phenomenological analysis (IPA) with thematic content analysis (TCA). As Frost [26] observes, the pluralistic approach enables flexibility “by building up multi-perspective layers of insight,” with each layer contributing to the understanding of the reality of those being studied. We used primarily IPA to explore the individuals’ lived experiences in depth, and to subsequently take the analysis to a broader level, where applicable, by using TCA to identify the broad themes that were emerging across the dataset. We conducted IPA as postulated by Smith and colleagues [27], in which the aim is to explore in detail participants’ life-worlds and the meanings they attach to these, in this case victimisation experiences of persons living with severe mental illness. The IPA has been found to be an appropriate method for analysing emotionally laden topics [28] pertinently including victimisation.

A detailed, case-by-case analysis was conducted by an experienced qualitative data analyst (first author) who read each of the transcripts iteratively to obtain a full picture of the participant’s experience of victimisation. While the analytical focus revolved around episodes of victimisation, both before and after onset of severe mental illness, analytical interest was also on issues that were found to have an influence on or were themselves influenced by victimisation. These were considered essential in constructing the participant’s entire story of victimisation and the meaning they made out of it, and included the participant’s life history, living conditions, family relationships, migration between households and livelihood activities. Within each individual’s narrative, nuances, patterns and themes were established. With the help of MS Excel, the analyst classified themes and sub-themes on a matrix, capturing each participant’s experiences (as reflected in verbatim quotes from the data) to illustrate the respective theme. These themes and sub-themes from each participant’s narrative/transcript were then discussed among team members before consensus was achieved. This was followed by the thematic content analysis phase, during which coding and analysis of the data was conducted across the individual cases with a view to identifying broader patterns and themes. This process also involved comparison of the themes from each individual’s narratives across the cases to establish consistencies and divergences, from which shared themes (those cutting across the different cases) were developed and illustrated by verbatim quotes from the data. The following three themes were discussed and eventually agreed upon by the research team as the key themes that constituted the essential character of the phenomenon of victimisation of persons with severe mental illness. They included psychological, physical and sexual victimisation.

### Validity and reliability

Validity in qualitative research is about accuracy of the findings, while reliability speaks to consistency of the analytical procedures irrespective of the researcher or dataset involved [29]. We promoted internal validity by recruiting and interviewing study participants until such a time that data saturation was achieved, a point at which no new information was obtained from additional interviewing. Additionally, external validity or transferability (the degree to which the findings from qualitative research are generalizable or transferable to other contexts) – was enhanced by providing a “thick description” [30] or full account of our methodological procedures, participants and findings. Thick description would allow the reader to assess the extent to which our inferences and conclusions are transferable to other settings. Relatedly, reliability was upheld through a systematic documentation of the research process from data collection to analysis, thereby creating an audit trail to enable other researchers to make informed judgments about the soundness of the process, and to replicate these as needed.

## Results

As mentioned earlier, three forms of abuse were evident from the data, including psychological (emotional), physical and sexual victimisation. Psychological victimisation – defined as “the infliction of anguish, pain, or distress through verbal or non-verbal acts… [and] includes but is not limited to verbal assaults, insults, threats, intimidation, humiliation, and harassment” [31] – was usually exhibited in the demeaning and rejection of persons with severe mental illness. Physical victimisation manifested in the form of beatings including forced indoor confinement and tethering, while sexual victimisation basically took the form of rape at the hands of either a member of the public or, notably, a relative.

In Table 1 we summarise the broader demographic characteristics of our participants. Overall, 12 of the 18 study participants reported victimisation while suffering from severe mental illness. One participant reported having experienced all the above three forms of victimisation; seven participants reported two of the three forms of victimisation; and five participants reported one of the three forms of victimisation. Furthermore, a total of eight participants reported a history of victimisation, with four of these subsequently being victimised in their later years while suffering from severe mental illness. Ten participants reported victimisation as having impacted their lives. It is worth noting that participants in this study were not stratified by gender, as we did not aim at the outset to explore gender – only five of a total of 18 study participants were male. This notwithstanding, some gender-based difference in the experience of victimisation could still be gleaned from the data, whereby only female participants reported sexual victimisation.

**Table 1:** Demographic characteristics of participants

Gender distribution	Male – 5 participants
	Female – 13 participants
Age distribution	< 25 years – 2 participants
	25 to 34 years – 6 participants
	35 to 49 years – 7 participants
	≥ 50 years – 3 participants
Socioeconomic status	Low – 8 participants
	Moderate – 10 participants
Mental disorder	Schizophrenia – 5 participants
	Bipolar affective disorder – 12 participants
	Major depressive disorder – 1 participant
Illness status at time of interview	All participants were in symptom remission

In presenting our key findings below, we primarily focus in depth on specific cases and later, on findings from our analysis across the cases. The specific cases were carefully selected to highlight the full spectrum of victimisation as reported by our study participants. Our purpose in taking this mainly case-intensive approach was to shed light on the context of victimisation, not just the act of victimisation, that is to say the circumstances or events leading to and following victimisation as the act of victimisation in itself is hardly sufficient in understanding the entire phenomenon of victimisation. But this detailed focus on a select few individual cases was also in keeping with phenomenology’s “idiographic sensibility” that is, a mindfulness of the uniqueness of participants’ life-worlds and, therefore, the need for an in-depth exploration of each case as a way of gaining a full understanding of the case, and only then seeking to establish patterns and themes across the cases [32, 33]. Moreover, the in-depth, case-intensive approach afforded us the ability to capture victimisation both in the context of severe mental illness and, where applicable, across the life course. The key themes and subthemes emerging from the data are summarised in Table 2 (below), and subsequently presented in more depth.

**Table 2:** Summary of key themes and subthemes presented in the results

• From an interpretative phenomenological analysis
− Psychological victimization
Attitudes towards mentally ill family members as valueless and dispensable
Stigmatization
− Physical victimization
− Sexual victimization
• From a thematic content analysis
− Failure to be understood by family members
− Differential experiences of victimization of women with severe mental illness

### Key findings from an interpretative phenomenological analysis

#### Psychological victimisation

Our analysis reveals that psychological victimisation was almost invariably blamed on the family – out of seven participants reporting this form of victimisation, six pointed to family as a source, with only two citing members of the public. Psychological victimisation manifested mainly in two ways. One of these was in attitudes towards mentally ill family members as valueless and dispensable. Two cases stood out in illustrating this type of psychological victimisation. One of the cases is that of Participant P1. This participant was a young woman in her second marriage. The marriage followed shortly after an earlier tumultuous marriage during which P1 gave birth to a baby at 15 years and conceived again eight months after delivery. This marriage would eventually end owing to her disagreement with the husband over his proposal to abort the second pregnancy. Although she was living with her parents at the time of this interview, P1 reported having developed mental illness during her second marriage, when she started “*walk[ing] through the streets of XX and all people got to know that [she] was mentally sick*.” She clarified having relocated from her matrimonial home purposely for the treatment of her mental health condition, and that she routinely left the matrimonial home every time she experienced a relapse of her mental illness. The participant shared about her run-ins with the current husband who at times verbally assaulted her with reminders of her mental illness, which “*made [her] feel so bad*.” It is these incidents, as P1 narrates, that eventually culminated in her husband asking her to go to her parents’ home for treatment during her latest illness episode. Yet, even at her parents’ home P1 still had distressful encounters with the caregiving mother:

Sometimes in the morning my next dose may be due, and I must take morning tablets. I may be in need of something to eat. She says there is nothing. So, instead of taking tablets twice a day, I only take for the night after getting something to eat…. Even when am sick she just tells me, ‘Go, buy poison and kill yourself’. Not that I always have it [money], but I may have Shillings10,000; she says ‘use it and buy poison and kill yourself’…. She means it! She tells me ‘Go, and buy… if you are to kill or not to kill yourself, don’t tell me. I have said go buy and drink it’… ‘if we surrendered you as a sacrifice, there is no problem’… I only want you people to talk to my mother on how to handle patients with mental illness. She gives me stress. She can tell me a word at a time when am sick and I hear something telling me to go and throw myself into a car [traffic].

As depicted in the above narrative, victimisation was apparent in terms of the patient’s psychological pain arising from a sense that her mental health condition was not being understood by the caregiver. Also notable in the excerpt is the victim’s allusion to the potential impact of the victimisation on prospects for her recovery and general wellbeing. This is particularly so when she expresses her perception of her mother rejecting and taunting her to end her life as a risk factor for suicide on account of its likelihood of triggering or coinciding with her hallucinations (the urge to throw herself into oncoming traffic).

What we read from the preceding excerpt is that victimisation can be uniquely impactful among some persons with severe mental illness. This is particularly so considering that the patient is dealing with a pre-existing health condition (severe mental illness) that primarily undermines their resilience or ability to adaptively navigate daily threats to their mental wellbeing. It was evident in this case that when such a patient encounters victimisation in any form, the impact may not always be attributable entirely to such victimisation but rather, to a synergy of factors, with victimisation only being an immediate cause.

Another case that illustrated attitudes of dispensability of mentally ill family members is that of P2, a female participant. This participant had been married for years and has children, but parted ways with the spouse on account of her mental illness. She also transferred her children to the care of her mother when she developed mental illness. At the time of this interview, P2 was living independently, and earned a living by engaging in trade. She shared about her life, highlighting significant historical events that could have led to her mental illness as well as her current experience with the mental illness and victimisation. Citing the former spouse as the abuser, she narrates about a fallout with him as a result of her mental health condition.

He [former spouse] married that woman like four years back…. Because right now they have three children. When I found him in the house, he told me: ‘omusajja tabeera n’abalalu’ meaning that ‘a man cannot live with a mad [mentally sick] person [wife]’. He told me to immediately leave his house. When he talked to me like that, I felt so stressed, and I relapsed and was brought back to hospital. Because of having very many thoughts about my husband, I would keep on relapsing; I was thinking about my children, I was not working at that time, it was the man [spouse] who used to look after us – so that made me relapse. After receiving treatment, I improved and was discharged. But on reaching home, now my children were looking up to me for food and all other requirements, which made me relapse again.

In this narrative, we observe that the participant was not only mistreated as worthless and dispensable because of her mental health condition but further asked to permanently leave her matrimonial home. Reflecting on this experience with the husband, the participant notably highlights her perception of a mutually reinforcing relationship between victimisation and mental illness. In this case, she attributes her victimisation – the psychological abuse of being denigrated by the husband and being evicted from her matrimonial home – to her mental illness, while at the same time blaming the victimisation for her (relapse in) mental illness. Victimisation, therefore, appeared to be perceived by the victim as resulting in a difficult-to-escape vicious cycle of mental illness. As well, the narrative brings to light the participant’s/victim’s sense of the multiple social factors that undermine recovery from mental illness, including poverty and related failure to take care of her offspring.

It is important to note that P2 not only encountered victimisation while suffering from severe mental illness; she also reported a history of sexual victimisation (rape) by the same man who would eventually marry her, only for him to ask her to leave their matrimonial home when she developed severe mental illness. In the following excerpt, for example, the participant narrates this history of victimisation and its impact, notably citing her mental illness as one such impact of being victimised.

When I think about that rape right now, I feel so bad…. I grew up not loving men. I was still a virgin. I had never loved any man [when I was raped]…[crying] … in fact [ever since that rape] I have never felt happy in my whole life [crying]. Not even on Christmas day have I ever been happy. That is the reason as to why I even became born again [crying]. Even after getting mental illness, I continued thinking about such abuses…. Those thoughts caused my mental illness because since that time I have never felt happy…. I still experience the thoughts about the abuse I suffered. [And] the person who would have comforted me would have been my husband, but we separated…. Whenever I think about this I cry.

Also remarkable is that the participant seemed to perceive the psychological victimisation – exhibited in her rejection by her former spouse – in more complex terms, not just in terms of the distress from being abandoned by a loved one because of a mental illness, which itself caused a series of relapses as reflected in her earlier narrative. The victimisation was also perceived in terms of denial by the spouse of a crucial form of (emotional) support that would have moderated the enduring psychological impact of the victimisation she had suffered in the earlier years (including rape) at the hands of the same man before the onset of her mental illness. We note, therefore, that the psychological pain the participant/victim suffered appeared to have been compounded by the ubiquity of the same man throughout her history of victimisation. This was not only the spouse who was rejecting her because of her mental illness, but he was the same man at whose hands she suffered rape several years before the onset of her mental illness. Indeed, we read from the victim’s account her sense that the very minimum the man behind her long history of victimisation, which culminated in mental illness, should have done was to provide emotional support.

Another way in which psychological victimisation manifested was stigmatisation. Two cases are featured to illustrate this type of abuse. One of which is that of Participant P3, a self-reliant, single young man who earned a living from selling produce from his own garden. This participant appeared to still be in denial about his condition when he disclosed: “*they [health workers] deceived me that it is mental illness…. I’m not very sure about my disorder*.” Nonetheless, P3 reported having been on medication for his mental health condition but admitted occasionally not adhering, which had caused relapses. It was noteworthy that the participant’s non-adherence had been reportedly driven by concerns about the drugs’ sedative effects (excessive sleep), which often kept him away from income-generating activities. We observed from P3’s narrative that this motivation to work hard arose at least in part from the poor relationship he had had with his relatives and, consequently, the frustration that no one was there to help him. He expresses this frustration with a proposal for how health workers could help with relatives.

It would be good for a health worker after treating someone with mental illness to also take time and call up his or her relatives and advise them on how to look after such a person. If a mentally ill person asks for new clothes, the caretakers should be able to provide them with such needs. If you [health workers] counsel them on how to handle mentally sick persons even those cases of beating up mentally sick persons will not happen.

While P3 responded rather adaptively to the anguish from the perceived lack of support from relatives by working hard to be self-sufficient and not have to seek support from elsewhere, he helplessly lamented the apparent psychological victimisation he encountered from community members who habitually stigmatised him because of his mental illness. These sometimes described him overtly in his presence as a mentally disturbed person.

You know where I stay people take me as a mentally disturbed person, that affects me a lot, some talk when you are hearing, and you really feel bad. I even had to forego attending church because of the same, I have spent nearly a year without going to church…. I am a Christian and I used to go to church since childhood, but it got to a point when I felt like God had forgotten me and as such I did not need to go to church.

As with all other cases, our interest in this case was in the experience but also the impact of such victimisation. We characterised this impact in two ways. First, we note that in withdrawing from church engagements the participant might have delinked and lost contact with some community members who constituted a potential source of social support networks. Second, and more profoundly, we observe that another impact of this victimisation might have been in P3 being prompted to question the essence of his very existence when, as highlighted in the preceding excerpt, he expressed having felt forgotten by God. This point will be better appreciated in light of the exceptionally high degree of religiosity in Africa [34]. In such a setting, therefore, where a relationship with God is usually of existential importance, the decision by P3 to stop attending church because of the victimisation was interpreted as the culmination of an existential crisis in which he felt abandoned not only by family and community but also, perhaps more importantly, by God.

The second case illustrating victimisation by way of stigmatisation was that of Participant P1. As cited earlier, P1 was a young, married woman who had relocated to her parents’ home and was living with the parents. Like her counterpart, P3, this participant was one of few cases who experienced victimisation both within the family and the broader community contexts. In addition to the victimisation she suffered at the hands of a family member who treated her as valueless and dispensable, P1 narrated about her encounters of victimisation by way of stigmatisation. She cited some man within the community who tried without success to take advantage of her vulnerability due to mental illness by renewing his previously unsuccessful sexual advances towards her after she developed mental illness.

What I see especially among men who are adults [is that] they usually use this opportunity – the fact that you are sick with mental illness [and that] maybe you will have low self-esteem and accept him, especially if you had refused him before…. Because he may think you have already lost hope. There is one that sweet talked me and I refused. Then he got my phone number and called, and he said, “no wonder you got mad.” So, I said to myself that let me not get discouraged because of his words, or quarrel with him.

The case of P1 was particularly intriguing, in that unlike the normal expectation that such victimisation would worsen their mental health condition or at least undermine their recovery process by keeping them in a constant state of stress and hopelessness, the participant in this case seemed to have a sense of purpose, that is, to beat the odds and maintain the path to her recovery from mental illness. She did this by fighting back against her abusers in resisting sexual victimisation and not allowing the stigma to impact her mental health condition negatively.

#### Physical victimisation

Unlike psychological victimisation, physical victimisation was almost equally blamed on the family and the public. Six of nine participants reporting physical victimisation cited a family member as the culprit, while four of the nine blamed the abuse on members of the community/public who included a traditional healer. As indicated earlier, physical victimisation manifested in beatings, indoor confinement and tethering. We present two cases to illustrate this type of victimisation. The first is that of Participant P4, a male participant aged over 55 years. This participant reported having never married, and that neither had he been involved in a relationship with a woman in a very long time. He also cited a history of victimisation by his parents and his teachers during childhood. Yet these experiences notwithstanding, the participant reportedly would eventually grow up to become a successful farmer who grew crops for both home consumption and the market. However, P4 lamented about his experience of physical victimisation, particularly by his own brother, when he developed mental illness.

I was tied up in chains and left to stay indoors for a period of two months. I ended up destroying my own house because I was scared of dying in the house…. It was my elder brother. He beat me up and this finger is lame as a result of being beaten by him. He took my property and tied me up in chains for quite some time. He would not provide me with drinking water and would give very little food, just enough for survival, which he would pour on a banana leaf placed on the floor. And I would ease myself in the same place.

This participant’s experiences of victimisation stood out in several ways. First, as evident in the above excerpt, the character of the physical victimisation itself was complex, involving not just beatings but also indoor confinement using chains, as well as denial of nutrition and hydration. Second, contrary to what might have been expected, the abusive brother appeared to have made no effort to have P4 access health care, in effect contributing to the deterioration of P4’s mental but also physical health. This might explain another peculiar occurrence, namely that it would take the initiative of P4’s friends rather than of family members for him to be able to access health care.

I had my friends from church who understood that I had a mental problem, so they brought me here at [name of health facility]. During those days, our doctor used to come from Butabika on every first Tuesday of a month. When he assessed me, he advised them to take me to Butabika for further assessments. They then took me to Butabika where I spent close to one month receiving treatment.

Reflecting on his experiences of victimisation following his mental illness, P4 appeared to be most concerned about the impact the abuse had had on his financial/economic security. He narrated about the economic setbacks he has suffered because of the illness.

All my plans for my financial welfare were ruined by relatives and the community members. I had a plantation of sugar canes, bananas, and many fruits such as jackfruits which were ransacked by people after knowing that I was receiving treatment from Butabika Hospital…. I have suffered a lot!! People always take my property. Whatever I try to do at home people steal it. They steal things like food crops and fruits. So that has made me lag behind in terms of financial stability.

Although financial victimisation did not feature prominently in our data, it was clear from the preceding excerpt that this form of abuse by notably family members, in synergy with the physical victimisation, might have to a considerable extent adversely impacted the trajectory of P4’s recovery and life in general. This is in light of the fact that, as the participant revealed, his property or resources that he would have needed to transition back to mental stability and normal life, including marrying and having children, was lost to his family members.

The second case featuring physical victimisation was that of Participant P5, an unmarried woman in her late 30s. Having dropped out of school because her parents could not afford school fees, this participant would later marry formally and start a family but did not have a child after almost a decade in marriage. The marriage eventually ended, and the former husband married another woman. At the time of this interview P5 was living with her grandmother. On how she earned a living, P5 shared about her involvement in cultivation on land owned by the grandmother, but also highlighted the difficulty she and her grandmother were going through in an effort to make ends meet.

The participant reported no history of victimisation, adding that her experience of being victimised was within the context of her severe mental illness in which the husband was the abuser. Indeed, as she revealed, while the termination of her marriage was against the background of years of marital disharmony over her inability to conceive, the husband’s decision to end the marriage was in fact prompted by P5’s mental illness. She described the abuse by the husband preceding the termination of the marriage:

He [husband] used a stick to beat me up…. He was asking me why I had burnt the clothes, he decided to beat me up. In fact, he first tied me up with a rope and then beat me up with a stick. After beating me up he then abused me sexually; he had tied my legs.

The participant went further to explain:

[When I fell sick] he should have looked after me, he should not have beaten me up. He beat me up and abused me sexually which made me hate him. At that time, I was not understanding properly – I had a small lamp which accidentally started a fire on a mosquito net and by the time I tried to put off the fire most clothes had been burnt except the bed sheets. As I finished rescuing the children most of the clothes had been burnt, but surprisingly when he came back instead of appreciating my efforts he just beat me up and abused me sexually.

In the preceding excerpt, the participant laments the failure by the husband to understand her condition as someone with mental illness, noting that she did not deserve the violence from the husband whose children she had rescued. She also highlights the immediate impact of her victimisation by the husband, including “*hatefulness*” towards him. Although she later added that she had got over this traumatic phase of her life and moved on, her lamentation in the same excerpt was indicative of the enduring psychological impact the victimisation had on her.

#### Sexual victimisation

Of the three forms of victimisation presented in this paper, sexual victimisation was the least reported, with only five of the 18 study participants disclosing having encountered this form of victimisation. All the victims of sexual victimisation were female. It was remarkable that two of these suffered the abuse at the hands of a family member. We present below Participant P6, one of the five cases of sexual victimisation. P6 pointed to a history of victimisation, intimating that as a young girl she was sexually victimised by a man who used to visit her aunt’s home where she lived. She was a middle-aged woman with adult children, and pregnant at the time of the interview. This participant’s situation was rather nuanced, in that she disclosed having separated with her husband and no more sexual contact with him for the last few months, but that the two still lived together. Moreover, the husband had already expressed a strong will to divorce P6 because he was reportedly “*fed up with [her] behaviour*.”

However, further analysis of data revealed that the baby that P6 was carrying might not have been the husband’s. Indeed, while the husband by implication acknowledged his paternity of the unborn child when he accompanied P6 to hospital and expressed to the health workers that he “*wanted us to stop on that number of children*,” P6 disclosed to the interviewer that the unborn child was her pastor’s. She further narrated the process leading to her sexual encounter with the pastor, revealing that she had sought counselling from him on how she might get her husband to accept conceiving one more child, but that instead the pastor visited her at home and engaged her in unprotected sex.

The participant also disclosed having had an earlier extramarital affair with another man when she and the husband lived separate lives but still shared a home. When rationalising the extramarital sexual relationship with the man, P6 depicted the husband as a man who was gradually abandoning his responsibilities and commitment to his wife. This depiction hinted at the thought process leading to her decision to accept the new man’s sexual advances, suggesting that her acceptance was, at least in part, the result of her perception of suboptimal care from her husband and the desire to make up for the shortfall in her care needs.

You see, for him he would use the trick of providing me with something to eat so I accepted [a sexual relationship with him] because I would be hungry, and I would want eats…. I would complain to him how my husband was making us to stay hungry during the day.

In this participant’s case, we observe the participant’s sense of victimisation by the husband by way of withholding assistance and marital commitment to his wife. But, despite what appeared like a consensual relationship as projected in the preceding excerpt, the participant also perceives victimisation by the man who was sexually exploiting her because of her vulnerability arising from her mental health condition and nutritional deprivation:

Sometimes he would want to force me – like when I was seven months pregnant, he forced me to have sex with him yet I had abdominal pains but he kept insisting on me having sex with him…. I felt very bad…. [He] used to force me against my own will…. Whenever I would ask him why he did it he would tell me that it was because he loved me so much, yet he had a wife, and I also had a husband.

In the context of P6’s severe mental illness and gaps in family support for mentally ill family members, we consider the victimisation by the two men who were sexually exploiting P6 as having been enabled by her husband’s abuse. This was reflected in her justification for her involvement with the men.

Interviewer: What do you think pushes you to have sex with other men, yet you are married?

Respondent: I think it is because they are helping you in one way or the other…. I think in one way or another those people are helpful to the mentally sick person.

In this case, the husband’s withholding of assistance to P6 appears to have created a support gap of which the men were taking advantage.

### Key findings from a thematic content analysis

#### Failure to be understood by family members

One of the key findings from our thematic analysis of data across the different cases was the participants’/patients’ concern at the failure to be understood by family members. This concern was reflected in the narratives of all the cases presented except one (P3). Also of note was that the failure to be understood was cross-cutting, affecting both those patients in spousal and in blood relationships. We observed that the failure to be understood constituted an important source of stress on the part of the patients. It was also noted that all the cases reflecting this form of victimisation, except one, were females. Failure to be understood by family members emerged as a key factor undermining prospects for recovery, as already presented, often leading to physical violence and denial of basic necessities (food and water) by family members. Cases in point, presented in detail earlier, include P1, who appealed to the health workers to educate caregivers about how to handle mentally ill relatives. They also include P2, who lamented about the rejection by the spouse and his denial of even emotional support, in spite of him having raped her as a young girl and taken her for a wife. The other case is that of P5 who reported about the husband beating her up supposedly for not taking enough care to prevent a house fire when in fact, as she reasoned, she did her best given her mental health condition.

#### Differential experiences of victimization of women with severe mental illness

Another notable observation from our thematic analysis were the differential experiences of victimisation of women with severe mental illness. These differential experiences were based on the kind of relationship: we noticed that while all the mentally ill women who endured victimisation at the hands of blood relatives were still kept in the family fold and continued to receive some social care from the abusive family members, the women who suffered victimisation at the hands of a spouse were rejected by their spouses and made to leave their matrimonial homes. Cases in point, presented in detail earlier, include P1, who was victimised and ultimately asked by the spouse to leave their matrimonial home. However, when P1 moved in and started living with her mother, she still suffered victimisation at the hands of the mother but was notably tolerated. Another case is that of P2 who was also asked by the spouse to leave their matrimonial home after being told bluntly that a man cannot live with a mad wife, even though the man had raped her as a young girl and eventually took her for a wife. We note that the case of P6 yet again demonstrates differential experiences of victimisation of women with severe mental illness, with those in matrimonial homes more likely to encounter harsher treatment within the family than those living with blood relatives. Akin to the cases of P1 and P2, we observed in P6’s case perhaps the beginnings of the process that might lead to eventual termination of her marriage. This observation may be further appreciated in light of (i) her disclosure of the husband having stated that he wanted a divorce because he was tired of her behaviour, and (ii) the fact that the husband was restricting her food intake, which prompted her to accept extramarital sexual advances from other men. Our presumption was that the two still lived together following the husband’s calculated decision to delay the divorce process because she was carrying his baby.

## Discussion

In this paper, we set out to explore the lived experiences and meanings of victimisation of persons living with severe mental illness, including the history and the typical abuser, and to discuss their implications for care of the mentally ill. To explore these, we used an interpretative phenomenological approach during data collection, but adopted a pluralistic analysis featuring both interpretative phenomenological analysis (IPA) and thematic content analysis (TCA). Our aim was to leverage the strength of both analytical approaches, conducting IPA for depth and TCA for breadth in our understanding of the subject matter. With 12 of this study’s 18 participants reporting victimisation while suffering from mental illness, our findings build up on the large body of work that has been done globally [2–5, 11] in pointing to the high prevalence of victimisation among the mentally ill.

Literature on victimisation of the mentally ill within the mental health care system has been scarce, with the incidence of victimisation of patients in the mental health care settings remaining largely obscure [35]. However, the reverse has been true in contexts outside of the mental health care system in both high income countries [36] and LMICs [11, 37] where the perpetrators within the community have been extensively described, including intimate partners and other family members. Even then, our study contributes to the literature by illuminating the predominance of the family environment in perpetrating victimisation of the mentally ill in some sub-Saharan African contexts. This is especially so given that we were unable to identify any sub-Saharan African study reporting similar findings, but also in light of the depiction in the literature of the protective character of the family social system in the African setting [38]. Relatedly, our findings point to the importance of lack of social support within family and the victimisation associated with this in determining the recovery trajectory of persons with severe mental illness. Both the female patient who expressed the enduring psychological pain of being physically assaulted by the husband because of a house fire, and another who agonised about being habitually taunted by the caregiver to take her own life because she was too demanding, for example, alluded to this victimisation as negatively impacting their recovery. This finding echoes those from a systematic review by Rani and colleagues [39].

Our findings have also revealed some patterns of experience among women with mental illness that point to some notable intra-gender heterogeneities. The findings showed that mentally ill women living in matrimonial relationships were more prone to extreme forms of victimisation than those living with blood relatives. To the spouse, a woman’s mental illness was intolerable and justified her ejection from the matrimonial home, whereas to blood relatives, she was tolerated and, despite reports of victimisation of the mentally ill adult, the relatives always made room for her as a family member. Previous studies [40–43] have investigated the lived experiences of caregivers of the mentally ill but have also noted the “surprising” paucity of literature on this subject given the frequent occurrence of mental illness [40]. Our findings build on this literature by bringing to light the heterogeneities in the victimisation experiences of women, with important implications for care of the mentally ill.

On the other hand, by revealing men’s intolerance towards their mentally ill female spouses our findings lend credence to what Hailemariam and colleagues [44], in their rural Ethiopian study on gender, mental illness and marriage, categorise as “gendered experiences of marriageability.” By this the authors refer to the unique challenge mentally ill women encounter being in a spousal relationship owing to their incapacity to conform to gendered sociocultural obligations within marriage. Similarly, our findings are consistent with the preceding authors’ in pointing out the inability of a woman to sustain her marriage in the wake of her mental illness. Hailemariam and colleagues [44] have sought to explain this phenomenon, particularly in the sub-Saharan African context, from a normative cultural perspective. They observe the gendered double standards by which marital separation is culturally acceptable due to a wife’s mental illness, but unacceptable when it is the husband with the mental illness, because in the latter case society expects the woman to take care of the ill husband.

With regard to coping ability, our findings highlighted gendered experiences, with men depicting higher resilience and coping abilities. Nonetheless, we identified behaviour among men that might have resulted in loss of social support from at least some community members, as reflected in total withdrawal from community/church engagement on account of being overtly labelled a mentally ill person. This finding echoes those from previous studies [45, 46] in pointing to the importance of stigma in mental illness, insofar as complicating the recovery process even for those who may be responding well to treatment and regaining normal functioning.

Our findings also illuminated the important role of basic human needs – food, water, sex and others – in the management of mental illness and shaping the recovery trajectory. While these basic/physiological needs are fundamental to human survival and general well-being [47] our study shows that these are even more key to the stability and recovery of persons with severe mental illness. This was illustrated by the woman who endured sometimes painful extramarital sex in exchange for food, as her husband was restricting her access to food. Our findings are consistent with those from previous work such as that of Williams and colleagues [48] in pointing to the importance of meeting basic needs in improving the quality of life of persons with severe mental illness. Relatedly, the failure to be understood was repeatedly highlighted in different patients’ narratives as a source of stress, raising concerns about poor coping and treatment outcomes. This echoes findings from Gaillard and colleagues’ [49] study where misunderstood mental health patients expressed frustrations of being “an object to be fixed, treated like a child.” The authors recommended efforts to heighten understanding and improve therapeutic relationships as a means of enhancing care for the mentally ill.

### Implications for care

Strategies for addressing victimisation of persons with severe mental illness in Uganda need to be sensitive to the local realities, mainly the sociocultural practices and beliefs. They also need to reflect an understanding of mentally ill women as a heterogeneous group, with dissimilar experiences based on family environment and relationships. Necessarily, these strategies would adopt a holistic framework for mental health interventions, incorporating not just biomedical but also social determinants of mental health. An intervention framework incorporating social determinants would be predicated on the understanding that the environment in which individuals live and work influences their health outcomes [50]. Thus, interventions for addressing victimisation of the mentally ill would focus on improving familial relationships, and social support and a sense of belongingness both within the family and the broader community, as these and related interventions have been found to be protective against adverse mental health outcomes [51, 52]. Indeed, these protective factors are recognised as being embedded in the African way of life [52]. For their part, mental health professionals would need to standardise “victimisation” in their tools for the assessment and psychosocial management of persons with severe mental illness.

However, our findings support those from previous work [53, 54] in revealing that some environments in sub-Saharan Africa, especially in the rural settings, are not necessarily always protective with respect to mental health. With this in mind, we propose realistic interventions that take advantage of and strengthen the protective aspects of the cultural and family support system, while seeking to change those aspects with deleterious effects on the recovery and wellbeing of those with mental illness – such as beliefs/norms around dealing with a mentally ill wife.

## Conclusion

Victimisation of persons with severe mental illness is an important social problem in sub-Saharan African settings such as Uganda, where the family environment plays a predominant role in its perpetration. Victimisation of the mentally ill has been found to impact negatively on their recovery and general wellbeing, by triggering victims’ harmful responses including a heightened risk of suicide, social withdrawal, a sense of hatefulness and worthlessness and a predisposition to more victimisation. We propose a holistic framework for mental health interventions, incorporating not just biomedical but also social determinants of mental health, and targeted at improving familial relationships, social support and a sense of belongingness both within the family and the broader community.

### Limitations of the study

A limitation of this study was that we did not aim at the outset to explore gender, and consequently our sample was not stratified to ensure gender balance. With only five of a total of 18 study participants being male we feel constrained in making substantive inferences on gender. We therefore propose similar studies in Uganda and the broader sub-Saharan African region that explore gender in more depth, especially given that we were still able to observe from our data some gender-based differences in the experience of victimisation.

## Data Availability

The datasets generated and/or analysed during the current study are accessible at 10.17037/DATA.00002840.

## Abbreviations

SMI: Severe Mental Illness
LMIC: Low and Middle Income Countries
HIV: Human Immunodeficiency Virus
IDC: Infectious Disease Clinic
IPA: Interpretative Phenomenological Analysis
